# Psychische Belastung und Beanspruchung: Die Bedeutung der Valenz und der sozialen Realität. Anmerkungen zu Ferreira und Vogt (2021)

**DOI:** 10.1007/s41449-022-00321-x

**Published:** 2022-06-28

**Authors:** Norbert K. Semmer, Dieter Zapf

**Affiliations:** 1 Institut für Psychologie, Universität Bern, Fabrikstrasse 8, 3012 Bern, Schweiz; 2 Institut für Psychologie, Goethe-Universität Frankfurt/M., Frankfurt/M., Deutschland

Ferreira und Vogt (2021) haben sich zum Ziel gesetzt, ein Kategoriensystem für psychische (Fehl‑)Belastung und psychische Ressourcen zu entwickeln, das eine Grundlage für die Bestimmung von Grenzwerten für psychische Belastung liefern soll. Das Thema ist insofern hoch relevant als Unternehmen gehalten sind, Gefährdungen auch für die psychische Gesundheit nach Möglichkeit zu vermeiden bzw. so gering wie möglich zu halten (ArbSchG 2022). Die dafür erforderlichen Gefährdungsbeurteilungen erfolgen, sofern sie überhaupt durchgeführt werden, nicht unbedingt nach einheitlichen Massstäben (Bundesanstalt für Arbeitsschutz und Arbeitsmedizin (BAUA) 2014). Zwar liegen Verfahren vor, und manche davon beinhalten auch Bewertungen als „kritisch“ o. ä. – und somit auch Grenzwerte (BAUA 2014). Viele Fragen sind jedoch noch nicht ausreichend geklärt, und der Beitrag von Ferreira und Vogt versucht eine begriffliche Klärung und stellt ein Forschungsprojekt vor, mit dem das Konzept empirisch untermauert werden soll.

Wir finden es wichtig, diese Diskussion zu führen, nicht zuletzt im Hinblick darauf, dass beeinträchtigte psychische Gesundheit ein grosses, und zunehmendes Problem darstellt, wie man nicht zuletzt and den Gründen für Arbeitsabwesenheiten feststellen kann (Meyer et al. 2021). Massnahmen zur Gestaltung der Arbeit in einer Art und Weise, die der psychischen Gesundheit zuträglich ist, sind daher nötig und eine ständige Herausforderung für Unternehmen und die Arbeitswissenschaften.

Wir können und werden nicht auf alle Aspekte des Beitrags von Ferreira und Vogt eingehen. Vielmehr greift der folgende Beitrag einige Aspekte auf, die aus unserer Sicht für die weitere Diskussion von zentraler Bedeutung sind. Diese Aspekte beziehen sich a) auf Definitionsfragen, nicht zuletzt im Hinblick auf die „Neutralität“ der Begrifflichkeit und die Unterscheidung von Stressoren und Ressourcen; b) auf die Bedeutung der „sozialen Realität“, c) auf Klassifikationsfragen, und d) auf die Frage der Grenzwertbestimmung.

## Belastungen als Risikofaktoren

Dem Arbeitsschutz im Hinblick auf psychische Gesundheit liegt das Belastungs-Beanspruchungs-Konzept zugrunde, das Rohmert und Rutenfranz (1975) definiert haben. Grundlegend an diesem Konzept ist, dass es Belastungen als von außen einwirkende Gegebenheiten und Beanspruchung als Auswirkungen auf den Menschen unterscheidet. Das Modell impliziert, dass potenzielle Gefährdungen nur über Beanspruchungsfolgen ermittelt werden können, wie Ferreira und Vogt auch mehrfach betonen. Entsprechend kann eine Belastung auch nur dann eine Fehlbelastung sein, wenn sie mit Fehlbeanspruchungen korreliert. Dabei geht es nicht um jeweils individuelle Reaktionen – diese sind auch bei identischen Bedingungen individuell verschieden. Gefährdungen können nur über die mit bestimmten Bedingungen verbundenen Risiken i. S. v. Wahrscheinlichkeitsbeziehungen bestimmt werden.

Es muss also gezeigt werden, dass bestimmte Bedingungen wie etwa die Länge der Arbeitszeit mit einem erhöhten Risiko psychischer Beeinträchtigungen verbunden sind. Dies zeigen z. B. Raediker et al. (2006) für psycho-vegetative Beschwerden in Abhängigkeit von den wöchentlichen Arbeitsstunden oder Folkard und Lombardi (2006) für Verletzungen in Abhängigkeit von ununterbrochener Arbeit ohne Pausen. Solche Daten verringern die Abhängigkeit von subjektiven Einschätzungen, und entsprechende Datengrundlagen können für verschiedene Unternehmensbereiche bzw. Tätigkeiten, aber auch für verschiedene Gruppen (etwa nach Alter oder Ausbildung) erstellt werden. Ferreira und Vogt folgen diesem Ansatz, allerdings mit einem aus unserer Sicht unrealistisch hohem Anspruch. Ihr Modell „postuliert, dass aversive Belastungsfolgen für *nahezu alle* Personen einer vorab definierten menschlichen Zielpopulation zur Fehlbelastung und damit zur Gefährdung werden“ (S. 4; Hervorhebungen NKS/DZ). Tatsächlich wirken sich Gefährdungen so gut wie nie für „nahezu alle“ in gleicher Weise aus.

So ist Rauchen als Risiko für Lungenkrebs inzwischen wohl unbestritten, aber „Risiko“ bedeutet dabei, dass Rauchende gegenüber nicht Rauchenden ein erhöhtes Risiko aufweisen, nicht aber ein in absoluten Zahlen hohes Risiko. Denn auch bei den Rauchenden stellen diejenigen, die an Lungenkrebs erkranken, eine Minderheit dar: Nach Drings et al. (2008) steigt das Risiko für ständig Rauchende von ca. 5 % im Alter von 65 auf 15,9 % im Alter von 75. Auch eine COVID-19 Infektion könnte nach dem Kriterium „fast alle“ nicht als Risikofaktor für Mortalität gelten.

In Übereinstimmung mit diesen Überlegungen berichten Li et al. (2020) ein relatives Mortalitäts-Risiko von 1,17 (95 % CI 1,05–1,31) für Arbeitszeiten von ≥ 55 h; sie betrachten diesen Zusammenhang als „moderate and clinically meaningful“ (S. 2). Das heißt: Belastungen sollten als Risikofaktoren verstanden werden, und Gefährdungsbeurteilungen sollten sich an solchen Effektgrössen orientieren. Am Rande sei diesbezüglich erwähnt, dass Effekte, die in Korrelationskoeffizienten ausgedrückt werden, häufig unterschätzt werden und relativ kleine Koeffizienten mit durchaus bedeutsamen relativen Risiken verbunden sein können; so kann ein Koeffizient von r = 0,19 zwischen mittels externer Beobachtung erhobenen Stressoren und psychosomatischen Beschwerden mit einem relativen Risiko von 3 verbunden sein (Frese 1985; s. a. Rosenthal 1990).

## Neutralität versus Valenz

Ferreira und Vogt legen grossen Wert auf begriffliche Klärung, und angesichts der durchaus uneinheitlichen Begriffsverwendung erscheint dies auch als sinnvoll. Zugleich verweisen ihre begrifflichen Vorschläge – nicht zuletzt die Verwendung der Begriffe *Fehlbelastung* und *Ressourcen* – auf Probleme, die mit der Neutralität der Begriffe Belastung und Beanspruchung verbunden sind. Der neutrale Belastungsbegriff spiegelt die Tatsache wider, dass der menschliche Organismus auf äussere Einflüsse reagiert, unabhängig davon, wie diese Einflüsse auf der einen und die Reaktionen auf der anderen Seite im Einzelnen aussehen. Er verweist auch darauf, dass Belastung nötig ist, um die Funktionsfähigkeit des Organismus zu gewährleisten und zu trainieren und dass somit die Abwesenheit von Belastung kein sinnvolles Ziel sein kann. Ein neutraler Belastungsbegriff kann also als Rahmenkonzept durchaus sinnvoll sein (wenngleich angesichts der im täglichen Sprachgebrauch vorherrschenden negativen Konnotation des Begriffs der Belastung schwierig); im Hinblick auf einzelne Aspekte eines solchen Rahmenkonzepts kommt man aber wohl nicht darum herum, positive und negative Aspekte zu unterscheiden.

Das Belastungs-Beanspruchungskonzept steht in der Tradition von Selyes Definition von Stress als „unspezifische Reaktion des Organismus auf jede Anforderung“ (Selye 1981, S. 170). Es hat sich jedoch schnell herausgestellt, dass positive und negative Anforderungen nicht in gleicher Weise wirken (Zapf und Semmer 2004), und dass sich bestimmte Belastungssituationen i. S. d. Belastungs-Beanspruchungskonzepts (z. B. überlange Arbeitszeiten; Arbeitsplatzunsicherheit, Rollenkonflikt oder sinnentleerte Arbeit) durchaus als Belastungen im negativen Sinn, also als Stressoren klassifizieren lassen. Diesem Umstand trägt der Begriff der *Fehlbelastung *Rechnung. Umgekehrt erweisen sich manche Umstände als *Ressourcen*, z. B. weil sie Lernmöglichkeiten bieten, soziale Kontakte ermöglichen u. ä. Selye hat dem dadurch Rechnung getragen, dass er die Begriffe des „guten“ und „schlechten“ Stress (Eustress und Distress) einführte.

Beanspruchungsreaktionen und -folgen hängen somit einerseits von Höhe und Dauer der Belastung ab, wobei geringe oder sogar positive Wirkungen ab einer bestimmten Höhe in negative Effekten umschlagen können, etwa im Hinblick auf die Dauer der Arbeitszeit. Die Beanspruchungsreaktionen hängen aber eben auch von der Qualität der Belastung ab. So haben hohe Anforderungen unterschiedliche Auswirkungen, je nachdem, ob sie für sinnvoll und legitim gehalten werden oder nicht (Semmer et al. 2019; Semmer und Zapf 2019). Zudem können hohe Anforderungen einerseits zu Stress führen, jedoch zugleich positive Effekte haben, wenn ihre Erfüllung Möglichkeiten zur Bestätigung des eigenen (beruflichen) Selbstbildes bietet (challenge stressors; LePine et al. 2005; Widmer et al. 2012). Dieser Bedeutungs-Aspekt spielt im Belastungs-Beanspruchungskonzept eine eher untergeordnete Rolle, obschon Rutenfranz bereits 1981 einen wesentlichen Bedeutungsaspekt, nämlich „Sinnentleerung der Arbeit“ (S. 389) hervorgehoben hat. Auch hier gilt selbstverständlich, dass es sich um eine probabilistische Beziehung handelt, die für verschiedene Individuen durchaus unterschiedlich sein kann.

In diesem Zusammenhang muss insbesondere die Rolle sozialer Stressoren betont werden, worauf auch Ferreira und Vogt verweisen. Soziale Stressoren spielen insofern eine spezielle Rolle, als sie nicht unbedingt mit Anforderungen verbunden sein müssen. Eine abschätzige Bemerkung stellt für die betroffene Person häufig nicht deshalb einen Stressor dar, weil sie auf eine bestimmte Art reagieren und dafür Energie aufwenden muss. Vielmehr ist es die abschätzige *Bedeutung*, die die Bemerkung zum Stressor macht. (Der Umgang mit dem dadurch ausgelösten Stress hingegen ist dann eine eigene Anforderung, wie etwa die Forschung zur Emotionsarbeit zeigt [Zapf et al. 2021], jedoch ist diese Anforderung nicht die Ursache, sondern die Folge des Stresserlebens; s. Semmer und Zapf 2019).

## Klassifikation von Stressoren und Ressourcen

Die Unterscheidung nach Valenz ist wichtig, aber sie stellt nur den Anfang dar. Man kann ja nicht davon ausgehen, dass die beteiligten Mechanismen für jede Art von negativer Beanspruchung (Stress) als Folge einer Fehlbelastung (Stressor) dieselben sind, und das gilt analog für Ressourcen. Somit stellt sich die Frage einer sinnvollen Klassifikation von Stressoren und Ressourcen.

Die im Rahmen des Arbeitsschutzes betonten Aspekte beziehen sich vor allem auf körperliche Aspekte der Beanspruchung, etwa durch Dauer und Lage der Arbeitszeit (s. Wong et al. 2019) oder durch Umgebungsbelastungen wie Lärm, Hitze, schlechte Ergonomie, etc. (Schlick et al. 2018).

Die Ergänzung durch den Aspekt der *Bedeutung* erfordert jedoch darüber hinaus eine Einordnung im Hinblick auf – im weitesten Sinne soziale – menschliche Bedürfnisse (s. Deci et al. 2017; Heckhausen und Heckhausen 2018; Talevich et al. 2017). Dazu gehören etwa das Bedürfnis nach Kompetenz, nach Möglichkeiten der Einflussnahme (Kontrolle, Autonomie), nach sozialer Eingebundenheit (Anschlussmotiv; Belongingness), wobei nach Ansicht vieler Autorinnen und Autoren dem Bedürfnis nach sozialer Bestätigung eine zentrale Rolle zukommt (Kruglanski et al. 2022: „social worth“; Leary und Allen 2011: „relational value“).

Damit lassen sich viele Aspekte der Arbeitsgestaltung einordnen, so etwa Arbeitsaufgaben, die bedeutsam sind, vollständige Handlungen ermöglichen, Abwechslung bieten, Autonomie ermöglichen und Feedback über den Handlungsverlauf und das Ergebnis beinhalten (Hackman und Oldham 1980; Schaper 2019; Ulich 2011); nicht zufällig wird im Job Characteristics Model von Hackman und Oldham (1980) die Wirkung von Bedeutsamkeit, Ganzheitlichkeit und Variabilität unter anderem (und gemäss Humphrey et al. 2007, vor allem) durch die erlebte Sinnhaftigkeit (meaningfulness) vermittelt. Aber auch der (befürchtete oder reale) Verlust des Arbeitsplatzes, der neben den materiellen Folgen nicht zuletzt die Möglichkeiten eines sinnvollen Beitrags zum und einer adäquaten Teilnahme am gesellschaftlichen Leben gefährdet (Mohr 2010; Sverke et al. 2002), ist hier zu erwähnen.

Hier spielt also nicht zuletzt die soziale Bedeutung der jeweiligen Tätigkeit und der damit verbundenen Aufgaben und Ausführungsbedingungen eine Rolle (vgl. das Konzept der „dirty work“; Kreiner et al. 2006). Vielfach wird die soziale Bedeutung nicht zuletzt darin sichtbar, dass eine als neutral oder gar positiv empfundene Tätigkeit zum Stressor wird, wenn sie als Resultat einer unfairen Zuteilung oder einer vermeidbaren Missachtung der eigenen Bedürfnisse und des eigenen beruflichen Selbstverständnisses angesehen wird (Semmer et al. 2019), wenn sie mit geringer Autonomie verbunden ist (Karasek und Theorell 1990), oder wenn den Anforderungen keine ausreichenden Belohnungen gegenüber stehen (Siegrist und Dragano 2020).

Auf der unmittelbar sozialen Seite geht es um soziale Stressoren (Gerhardt et al. 2021), d. h. um soziale Abwertung (relational devaluation; Leary und Allen 2011) durch das Verhalten anderer. Das reicht von unhöflichem Verhalten (Incivility; Cortina et al. 2017), das im Einzelfall möglicherweise harmlos, aber in der Häufung potenziell ausserordentlich stressauslösend ist, über sozialen Ausschluss, Diskriminierung, Beleidigung bis hin zum Mobbing/Bullying (Einarsen et al. 2020) und zu körperlicher Aggression. Auch die damit typischerweise verbundenen Emotionen und der (gewünschte/geforderte/erlaubte) Umgang mit diesen Emotionen (Emotionsarbeit/Emotion-rule Dissonanz: Hülsheger und Schewe 2011; Zapf et al. 2021) wären hier zu nennen.

Nicht alle Handlungen stehen unmittelbar in Zusammenhang mit grundlegenden Bedürfnissen. Ziele sind hierarchisch organisiert (Kruglanski et al. 2002; Hacker 2020), und einzelne Handlungen oder Handlungsschritte sind nur mittelbar – über das Erreichen der jeweiligen Handlungsziele – mit den grundlegenden Bedürfnissen verbunden. Allerdings ist nicht nur der instrumentelle Charakter dieser Ziele, also die erwartete Befriedigung der übergeordneten Ziele, relevant für die Beanspruchung. Die Zielerreichung selbst, ja sogar die Annäherung an diese Ziele, ist im positiven Fall motivierend und mit positiven Emotionen verbunden und kann somit als Ressource wirken (Grebner et al. 2010); umgekehrt ist die Zielverfehlung bzw. die ungenügende Ziel-Annäherung ein Stressfaktor (Plemmons und Weiss 2013; Carver und Connor-Smith 2010).

Aus dieser Perspektive sind Klassifikationen, die sich mit Regulationshindernissen beschäftigen, relevant. Diese äussern sich z. B. in „arbeitsorganisatorischen Problemen“ (performance constraints) (s. Irmer et al. 2019; Zapf und Semmer 2004) oder im Problem der Unterbrechungen (Baethge et al. 2015; Keller et al. 2020).

Insgesamt ergeben sich somit drei grosse Klassen von Faktoren: a) körperlich (Anstrengung, physikalische Einflüsse), b) Aufgabenmerkmale (Abwechslung, Ganzheitlichkeit, Kontrolle), c) unmittelbare soziale Einflüsse (Konflikt, Unhöflichkeit; Mobbing, etc.). Viele Instrumente zur Gefährdungsbeurteilung beinhalten solche Aspekte (BAUA 2014), wenngleich mit unterschiedlicher Schwerpunktsetzung. Allerdings können die jeweils spezifischen Sub-Aspekte in solchen Verfahren nur bedingt berücksichtigt werden, und derselbe Wert kann an verschiedenen Arbeitsplätzen auf jeweils unterschiedlichen Merkmalen beruhen. Beispielsweise können arbeitsorganisatorischen Problemen Qualitätsmerkmale von Material, von Werkzeugen, oder von Information zugrunde liegen, soziale Stressoren können aus Interaktionen mit Kolleginnen und Kollegen, Vorgesetzten oder KundInnen erwachsen (Dormann und Zapf 2004). Solche Aspekte im Detail zu berücksichtigen, wäre nur in sehr umfangreichen oder in arbeitsplatz-spezifischen Instrumenten möglich, was im ersten Fall Instrumente mit einem hohen Anteil an „trifft nicht zu“ oder ähnlichen Antworten ergibt und im zweiten Fall die Vergleichbarkeit der Instrumente einschränkt. Wir sind hier skeptisch und gehen davon aus, dass die Detailarbeit durch die jeweiligen Teams von Beteiligten und Expertinnen wohl unumgänglich ist (s. unten). Deren Einschätzung ist auch speziell wichtig für die Beurteilung der sozialen Aspekte von Aufgabenmerkmalen (z. B. der Legitimität von Autonomie-Einschränkungen auf Grund hoher Sicherheitsanforderungen).

## Subjektivität und soziale Realität

Das Belastungs-Beanspruchungskonzept steht in einer medizinisch-naturwissenschaftlichen und ingenieurwissenschaftlichen Tradition, was beispielsweise bei Rutenfranz (1981) und den dort berichteten Beispielen deutlich wird. Das Bemühen um Objektivierung ist deutlich, und naturwissenschaftliche Messmethoden spielen eine zentrale Rolle. Psychische Aspekte zu messen, gilt daher vielfach als schwierig bis unmöglich, wenngleich in der neueren Literatur Phänomene wie Resilienz, Burnout und Mobbing als in das Belastungs-Beanspruchungsmodell einfügbar diskutiert werden (Schlick et al. 2018). Die Phänomene selbst werden also durchaus ernst genommen, die Betonung der Subjektivität ist aber mit grosser Skepsis im Hinblick auf „Objektivierbarkeit“ verbunden. Und in der Tat ist die Rolle der subjektiven Bewertung (in der Psychologie spricht man von „Appraisal“; Lazarus 1999) bei psychischen Phänomenen besonders wichtig, denn viele psychische Belastungsfaktoren führen vor allem dadurch zu negativer Beanspruchung (Stress), dass sie als Bedrohung für das eigene Wohlergehen empfunden werden (Lazarus 1999), woraus eine negative Anspannung resultiert (Zapf und Semmer 2004). Für diese Interpretation ist es nicht nötig, dass biologisch definierte Ressourcen (z. B. Energie) übermässig in Anspruch genommen werden: Vielfach ergibt sich das Appraisal als „belastend“ schlicht aus der sozialen Bedeutung, die Ereignisse oder Bedingungen haben (Semmer und Zapf 2019). Ein als unfair empfundenes Feedback beispielsweise muss nicht mit neuen Anforderungen verbunden sein – die mangelnde Fairness genügt für die Einschätzung als belastend, weil damit das Bedürfnis nach Anerkennung und Zugehörigkeit verletzt wird (s. Krings et al. 2015). Dasselbe gilt für viele soziale Stressoren, so z. B. respektloses oder beleidigendes Verhalten (Gerhardt et al. 2021).

Mit Skepsis ist dieser Sachverhalt wohl nicht zuletzt deshalb verbunden, weil die subjektive Bewertung mit *individueller* Bewertung gleichgesetzt und dadurch nicht als „objektive Realität“ anerkannt wird (s. Semmer und Zapf 2019). Diese Betrachtung vernachlässigt jedoch, dass es auch eine *soziale* Realität gibt, und dass diese durchaus objektiviert werden kann. Schliesslich sind Kulturen „shared meaning systems“ (Erez 2010). Sie stellen die Basis für die Bewertung organisationaler Praktiken im Hinblick auf die eigene Person und das eigene Wohlbefinden dar (Gelfand et al. 2017). Das schliesst nicht zuletzt ein, dass auch Emotionen und die ihnen zugrundeliegenden Bewertungsprozesse kulturell mitbedingt sind (Ashforth und Humphrey 2022; Tsai 2017; van Kleef et al. 2016). Der Kulturbegriff darf dabei nicht auf nationale Kulturen eingeengt werden. Kulturen existieren auf vielen Ebenen; sie können regionaler, religiöser, sprachlicher, berufsbezogener, statusbezogener usw. Natur sein (s. Adler und Aycan 2018; Ashforth et al. 2020; Gelfand et al. 2017). Entscheidend ist dabei, inwieweit man sich mit sozialen Einheiten auf verschiedenen Niveaus identifiziert, also die „soziale Identität“ (Hogg und Terry 2000). Somit kann für bestimmte, identifizierbare soziale Einheiten – repräsentiert beispielsweise durch den Beruf, den Status, die Zugehörigkeit zu einer bestimmten Organisation, usw. – eine gewisse kulturelle Prägung von Interpretations- und Bewertungsprozessen angenommen werden. Dies führt selbstverständlich nicht zu vollständiger Homogenität, es bleiben immer noch Unterschiede, die sich aus individuellen Eigenheiten, der Zugehörigkeit zu Subgruppen oder der simultanen Zugehörigkeit zu anderen sozialen Einheiten (Geschlecht, Herkunft) o. ä. ergeben (Ashforth et al. 2020). Aus ihrer sozialen Prägung ergibt sich jedoch, dass Interpretations- und Bewertungsprozesse in Bezug auf belastende (hier gemeint als: stressinduzierende) Aspekte der Arbeit nicht nur individuelle Prozesse sind; sie sind vielmehr durch solche soziale Zugehörigkeiten mitgeprägt – diese konstituieren eine „soziale Realität“, die durchaus mehr Objektivität der Messung ermöglicht, als man bei einer rein individuellen Betrachtung annehmen würde.

Auf der Basis der Kenntnis dieses Kontexts ist es dann durchaus möglich, Stressoren (Fehlbelastung) objektiv zu erfassen, wobei objektiv bedeutet: unabhängig von der jeweils *individuellen* Reaktion (Semmer et al. 2004; Sonnentag und Frese 2013; Sonntag und Feldmann 2018). Der Zusammenhang zwischen Stress-Indikatoren und Stressoren bei Frese (1985) beruht beispielsweise auf Beobachtungen durch externe Expertinnen und Experten, und auch andere Untersuchungen verwenden Beobachtungsdaten (z. B. Elfering et al. 2018b; Grebner et al. 2005; Greiner et al. 2004; Karstad et al. 2018; Semmer et al. 1996) oder die Einschätzung anderer Personen, wie etwa Kolleginnen und Kollegen (Pindek und Spector 2016) oder Vorgesetzte und Partnerinnen/Partner (Meier und Semmer 2018). Dabei lässt sich im Prinzip auch die *Bedeutung* bestimmter Umstände oder Verhaltensweisen erfassen, die diese erst zu Stressoren werden lässt – etwa, wenn externe Beobachterinnen und Beobachter beurteilen, ob ein bestimmtes Verhalten als unfreundlich klassifiziert werden kann (Keller et al. 2019). Diese Logik ist uns etwa aus dem Justizwesen durchaus vertraut, z. B. wenn das Verhalten einer Person als objektiv diskriminierend klassifiziert wird. Dies zeigt, dass diese Vorstellung von Objektivität im gesellschaftlichen Leben durchaus verankert ist. Auch hier liegen letztlich – in erheblichem Masse geteilte – subjektive Einschätzungen zugrunde, die normative Wirkung entfalten.

Subjektive Einschätzungen sind nicht zuletzt deshalb „verdächtig“, weil sie von einer Vielzahl von Faktoren abhängen, die mit den Arbeitsbedingungen nicht viel zu tun haben (wobei allerdings oft vernachlässigt wird, dass das auch für viele „objektive“ Messmethoden gilt; Semmer et al. 2004). Insbesondere die „negative Affektivität“, also die generelle Neigung, negative Emotionen zu empfinden, spielt hier eine grosse Rolle (Semmer und Meier 2009). Solche Einflüsse sind vor allem dann problematisch, wenn es um individuelle Aussagen der betroffenen Personen geht („self-report“); allerdings gibt es durchaus Möglichkeiten, solche Einflüsse statistisch zu kontrollieren. Längsschnitt-Daten zeigen, dass (selbstberichtete) Stressoren in der Arbeit auch dann verschlechtertes Befinden vorhersagen, wenn das Ausgangsniveau des Befindens kontrolliert wird (z. B. Igic et al. 2017).

Vor allem eine Möglichkeit, subjektive Einschätzungen zu objektivieren, wird zu wenig genutzt. Wenn mehrere Personen Arbeitsbedingungen einschätzen, ist der Durchschnitt dieser Einschätzungen deutlich weniger von den individuellen Befindlichkeiten und möglichen Verzerrungen beeinflusst. Frese (1985) zeigt, dass der Median individueller Einschätzungen der Arbeitsbedingungen durch mehrere Arbeiter mit derselben Tätigkeit mit dem individuellen Befinden zusammenhängt.

So kann man Gruppen von Arbeitenden mittels „Multilevel Studien“ (Hox et al. 2017) untersuchen und auf der Gruppenebene (Level 2) erfassen, was die jeweiligen Mitglieder einer Gruppe *gemeinsam* haben, während auf Level 1 die je *individuellen* Abweichungen von den Gruppenwerten abgebildet werden. Sofern sich dabei ein Effekt auf Level 2 zeigt, spricht das für einen Effekt der belastenden Arbeitsbedingungen, denn die jeweiligen idiosynkratischen Prozesse sind kontrolliert (allerdings werden damit auch objektiv vorhandene Unterschiede zwischen Individuen zu den idiosynkratischen Effekten gezählt, etwa wenn Mitarbeitende mit weniger Seniorität mit älteren Computern arbeiten müssen).

Mit einer solchen Multilevel-Analyse konnten Ortíz-Bonnin et al. (2021) zeigen, dass die Angaben von Hotel-*Teams* zu „Emotion-rule Dissonanz“ (wie häufig muss man Emotionen zeigen, die man in der Situation normalerweise nicht hat?) mit Kundenzufriedenheit zusammenhingen; die individuellen Einschätzungen (Level 1) waren dabei kontrolliert. Eine vergleichbare Multilevel-Analyse berichten Escartín et al. (2021) in Bezug auf Mobbing. Semmer et al. (1996) zeigen, dass man Stressoren als latente Variable modellieren kann, für die die Einschätzungen von zwei Arbeitern mit derselben Tätigkeit sowie von zwei Beobachtern/Beobachterinnen als Indikatoren verwendet wurden, wodurch die je individuelle Subjektivität der Einschätzungen keinen Einfluss mehr hat. Auch für das Befinden der Beschäftigten wurde eine latente Variable „shared job strain“ gebildet, mit den Werten der zwei Beschäftigten als Indikatoren. Die latenten Stressoren korrelierten deutlich mit dem „shared job strain“.

Vorgehensweisen wie Gruppenbildung oder der Einbezug von Beobachtungsdaten, Experteneinschätzungen oder Urteile von Kolleginnen und Kollegen ermöglichen also eine Objektivierung subjektiver Einschätzungen, und sie können die „geteilte Subjektivität“, die kulturelle Normen widerspiegelt, mit einbeziehen.

## Zur Frage der Grenzwerte

Ferreira und Vogt wollen mit ihrem Projekt Grundlagen für Grenzwerte liefern. Hier stellt sich die Frage, inwieweit wissenschaftliche Untersuchungen überhaupt Grenzwerte bereitstellen können. Ferreira und Vogt verweisen auf die Deutsche Gesetzliche Unfallversicherung (DGUV 2013), die damit argumentiert, dass ein einzelner Belastungsfaktor oft keine Gefährdung darstellt und dass über deren Zusammenwirken zu wenig bekannt ist. Ferreira und Vogt verweisen jedoch auch darauf, dass dies bei physischen Belastungsfaktoren nicht anders ist.

Als erste Annäherung bietet sich hier die Verwendung von Gesamt-Indices an. Sie hat sich durchaus bewährt – sei es als Gesamtskala mit den Einzelskalen als Items (Frese 1985), als Verhältnis aus Belastungen (Effort) und Ressourcen (Rewards; Siegrist und Dragano 2020) oder als Differenz aus den standardisierten Mittelwerten von Ressourcen und Stressoren (Elfering et al. 2018a). In allen Fällen zeigen sich deutliche Zusammenhänge zwischen dem jeweiligen Index und gesundheitsbezogenen Variablen. Solche summarische Indices können zumindest einen ersten Überblick liefern. Bei erhöhten Gesamtwerten ist dann natürlich angezeigt zu prüfen, welche Einzelwerte dafür insbesondere verantwortlich sind.

Was aber bedeutet das für Grenzwerte? Nach unserer Auffassung kann die Wissenschaft nur Zusammenhänge erforschen und auf dieser Basis Risiken definieren. Die Grenzwerte, die man daraus ableitet, sind in der Regel nicht im strengen Sinn „wissenschaftliche“ Grenzwerte, sondern vielmehr Grenzwerte, die auf einer wissenschaftlichen Basis beruhen. So sind die ermittelten Zusammenhänge zwischen Blutdruck und Krankheitsrisiko die Grundlage für die Grenzwerte für Bluthochdruck (Drings et al. 2008). Ab wann ein Risiko als zu hoch eingeschätzt wird, stellt eine gesellschaftliche Entscheidung dar. Solche Entscheidungen werden häufig von wissenschaftlichen Gremien getroffen, und das ist angesichts von deren Expertise auch sinnvoll. Man muss sich aber dessen bewusst sein, dass hier gesellschaftliche Entscheidungen getroffen werden, für die es eine wissenschaftliche Grundlage gibt, dass diese wissenschaftliche Grundlage aber in der Regel nicht *direkt* zu „wissenschaftlichen Grenzwerten“ führt.

Am einfachsten ist die Bestimmung von Grenzwerten, wenn es einen Punkt gibt, an welchem ein System kippt. Stresstheorien legen das ja durchaus nahe (Semmer und Zapf 2018). Man geht davon aus, dass eine geringe bis mittlere Belastung noch gut bewältigbar ist. Aber ähnlich wie in der Materialforschung ist irgendwann ein Punkt erreicht, an welchem die Belastungshöhe die Leistungsvoraussetzungen der Person kritisch übersteigt und es dadurch zu einer deutlichen Verschlechterung von gesundheitlichen und/oder leistungsbezogenen Parametern kommt. Beispielsweise zeigen Chandola et al. (2006), dass eine Konstellation nach dem erweiterten Karasek-Modell (ISO-Strain) erst dann die Entwicklung des metabolischen Syndroms signifikant vorhersagte, wenn sie in mindestens drei (von vier) Messungen im Verlauf von 14 Jahren auftrat.

Abgesehen davon, dass viele Zusammenhänge durchaus linearer Natur sind, sind jedoch nonlineare Effekte auch dort, wo sie existieren, nicht leicht zu finden. Zum einen zeigen sie irgendwann Wirkungen, die für die Unternehmen wirtschaftlich relevant sind – schliesslich sind Effekte auf Leistung, Fehlzeiten, Kündigungen usw., inzwischen durch eine Reihe von Meta-Analysen gut belegt (z. B. Gilboa et al. 2008; Gerhardt et al. 2021; Hülsheger und Schewe 2011); darauf reagieren Unternehmen oft mit Veränderungsmassnahmen. Zum anderen zeigt sich der sog. „healthy worker effect“, d. h. die Mitarbeitenden verlassen die belastende Situation (vgl. für den Bereich Schichtarbeit Frese und Semmer 1986). Man wird dann also auch in dem Bereich, in welchem die Zusammenhänge noch weitgehend linear sind, einen cut-off Punkt bestimmen müssen. In vielen Fällen liegt es dann nahe, eine statistische Größe zu wählen, z. B. eine Standardabweichung über (bei Stressoren) bzw. unter (bei Ressourcen) dem Mittelwert.

Die Diskussion um solche Grenzwerte ist natürlich immer auch Gegenstand von Interessenskonflikten. Durch die wachsende Evidenz dafür, dass ungünstige psychosoziale Arbeitsbedingungen mit gesundheitlichen Risiken, aber auch betrieblichen Kosten einhergehen und dass daher der Abbau von Stressoren und der Aufbau von Ressourcen ein Anliegen aller betrieblichen Interessensgruppen sein sollte, könnten sich diese Interessenskonflikte aber entschärfen.

In der jeweiligen betrieblichen Praxis aber kommen mehrere Faktoren hinzu: Es wirken immer gleichzeitig eine Vielfalt von Belastungen und Ressourcen, es gibt individuelle Unterschiede, und es gibt Unterschiede in den je individuellen Bedingungen bei im Prinzip vergleichbaren Tätigkeiten. Das setzt Grenzen für allgemeine Grenzwerte und spricht dafür, dass beispielsweise Expertinnen und Experten, die in diesen Dingen ausgebildet sind, für ein Unternehmen oder einen Unternehmensbereich festlegen, ob die Gesamtbelastung so hoch ist, dass die Risiken für psychische Beeinträchtigungen das Zumutbare überschreiten. An die Ausbildung dieser Expertinnen und Experten wären hohe Anforderungen zu stellen, und es müsste sichergestellt werden, dass etwa die Arbeitsmedizin, die Arbeitswissenschaft und die Arbeitspsychologie dort vertreten wären. Ebenso wäre sicherzustellen, dass auch die Betroffenen in solchen Gremien vertreten sind.

Eine solche Delegation an Expertinnen und Experten gibt es in vielen Bereichen. Ärztinnen und Ärzte entscheiden über die Arbeitsfähigkeit von Personen; Behörden können Betrieben Auflagen machen, Strassen sperren, Fahrzeuge auf dem Verkehr ziehen, usw., wenn sie Risiken für zu hoch halten. Sie dürfen nicht willkürlich entscheiden, sondern müssen Grundlagen beachten, die in vielen Fällen auf den Stand der Wissenschaft aufbauen, und sie müssen die Sicht der jeweils Betroffenen einbeziehen. Auf dieser Basis ist es aber ihr Gesamteindruck, der letztlich die Entscheidung prägt, weil nur dadurch die Gesamt-Konstellation berücksichtigt werden kann. Solche Entscheidungen können dann natürlich angefochten werden, z. B. vor einer Einigungsstelle.

In vielen Fällen wird bei betrieblichen Gefährdungseinschätzen tatsächlich so vorgegangen (z. B. durch den Einsatz von moderierten Analyseworkshops; BAUA 2014); manche Instrumente sehen entsprechende Analyseteams explizit vor (z. B. Sonntag und Feldmann 2018), und viele Instrumente sehen auch Bewertungen vor, die problematische Ausprägungen indizieren (z. B. Hacker et al. 1995; Sonntag und Feldmann 2018).

## Anknüpfungspunkte für die Forschungsfragen

Neben der Frage der Grenzwerte zielen Ferreira und Vogt vor allem auf die Frage, inwieweit ein berufsgruppenübergreifendes Kategoriensystem möglich ist. Das ist eine wichtige Fragestellung, aber man darf dabei nicht übersehen, dass dazu doch Einiges vorliegt. Kategorisierungen wie das Job-Characteristics Modell (Hackman und Oldham 1980), das Job Demands-Control (JD-C) Modell (Karasek und Theorell 1990) und dessen Erweiterung zum Job Demands-Resources (JD-R) Modell (Bakker und Demerouti 2017) beziehen sich auf eher allgemeine Kategorien, ebenso wie beispielsweise Siegrist’s ERI-Modell (Siegrist und Dragano 2020). Auch die Zusammenstellung verschiedener Stressfaktoren bei Sonnentag und Frese (2013) ist eher allgemein. Viele Instrumente beruhen auf Klassifikationen, z. T. auf spezifischer theoretischer Basis wie etwa der Handlungsregulationstheorie, die verschiedenen Instrumenten zugrunde liegt, wie etwa dem TBS (Hacker et al. 1995), ISTA (Semmer et al. 1999; Irmer et al. 2019), oder RHIA (Leitner et al. 1987) – s. dazu Hacker (1995), z.-T. auf breiterer Basis, wie etwa bei der „Gefährdungsbeurteilung Psychische Belastung“ (GPB; Sonntag und Feldmann 2018). Unabhängig von der jeweiligen theoretischen Grundlage sind in vielen Instrumenten die jeweiligen Konstrukte durch relativ wenige Items abgedeckt (so etwa auch im COPSOQ; Pejtersen et al. 2010; oder im WDQ; Stegmann et al. 2010).

Es gibt also viele Vorarbeiten, an die man anknüpfen kann. Ein Problem, das aus einer berufsübergreifenden Klassifikation erwächst, ist natürlich, dass diese notgedrungen eher wenige, und daher eher abstrakte Kategorien enthalten muss, die sich aber in unterschiedlichen Arbeitsfeldern sehr unterschiedlich äussern können. Die Alternative, Stressoren sehr spezifisch zu formulieren, würde zu endlosen und schwer handhabbaren Listen von Stressoren und Ressourcen führen, von denen jeweils grosse Teile für ein spezifisches Anwendungsfeld irrelevant wären (z. B. soziale Stressoren durch Kundinnen und Kunden/Klientinnen u. Klienten bei Tätigkeiten ohne solche Kontakte – s. oben, Punkt 3). Anzustreben wäre daher eine hierarchische Klassifikation, die auf einer höheren Ebene allgemeine Kategorien enthält (z. B. soziale Stressoren) und dann zunehmend spezifischer wird und beispielsweise auf Stress durch Klientinnen und Klienten Bezug nimmt, und dann auf Stress durch psychisch gestörte Klientinnen/Klienten usw. Auf den untersten Ebenen wird es aber wohl kaum möglich sein, jede spezifische Form eines bestimmten Stressors/einer bestimmten Ressource in eine Klassifikation aufzunehmen; hier sind dann die Gremien gefragt, die die Analysen durchführen und die dann die Aspekte beurteilen müssen, die sehr spezifisch für das jeweilige Anwendungsfeld sind (s. oben, Punkt 5).

## Schlussbemerkungen

Wir argumentieren in diesem Beitrag, dass Risiken, die von psychischen Belastungen ausgehen, dann objektiviert werden können, wenn man berücksichtigt, dass die Interpretation von Belastungen i. S. v. Stressoren oder Ressourcen kein rein individuelles Phänomen darstellt, sondern dass sich gesellschaftliche Normen herausgebildet haben, die diese Interpretationen prägen und die insofern eine soziale Realität darstellen. Diese soziale Realität lässt sich durchaus wissenschaftlich erfassen und etwa durch Urteile von Expertinnen und Experten und durch die Aggregierung individueller Urteile reliabel messen. Auf dieser Grundlage werden sich auch Grenzwerte für psychische Belastungen definieren lassen.

Die beteiligten Wissenschaften – insbesondere Psychologie, Medizin, Arbeitswissenschaft – sind gefordert, auf dieser Basis wissenschaftliche Grundlagen zu liefern, die über bislang dominierende Forschungsansätze hinausgehen, indem sie die Beurteilung vieler auf sinnvolle und reliable Weise verknüpfen und zudem die Wirkung *langfristiger* Expositionen erforschen. Die Schwierigkeiten, auf die man mit solchen Ansätzen stösst, sind allerdings erheblich. Beobachterinnen und Beobachter zu trainieren, ist ausserordentlich aufwendig, und die entsprechenden Kategoriensysteme zu konstruieren, erfordert viel Zeit und Aufwand (Greif et al. 1991; Karstad et al. 2018). Untersuchungen, an denen mehrere Personen mit vergleichbaren Tätigkeiten teilnehmen, sind schwierig, und so sind Untersuchungen, die Expositionen über längere Zeit vorsehen. Alles zusammen zu realisieren, stellt hohe Anforderungen an Forschungskompetenz, Aufwand und Finanzierung. Angesichts der Probleme, die mit psychischer Belastung verbunden sind – von individuellem Leid über Kosten für das Unternehmen bis zu den Belastungen für das Gesundheitssystem und den gesamtwirtschaftlichen Folgen – ist dieser Aufwand jedoch gerechtfertigt.
